# Catheter closure of a recanalized vertical vein after repair of total anomalous pulmonary venous connection

**DOI:** 10.1002/ccr3.1443

**Published:** 2018-03-11

**Authors:** Ramy Charbel, Najib Hanna, Linda Daou, Zakhia Saliba

**Affiliations:** ^1^ Department of Pediatrics Hotel‐Dieu de France University Medical Center Beirut Lebanon; ^2^ Department of Pediatric Cardiology Hotel‐Dieu de France University Medical Center Beirut Lebanon

**Keywords:** Catheter closure, congenital heart disease, total anomalous pulmonary venous connection, vertical vein

## Abstract

The vertical vein is sometimes left open in repair of total anomalous pulmonary venous connection. It usually closes later but can remain patent leading to a significant shunt. We describe a recanalized vertical vein in a 7‐year‐old having undergone repair in infancy. It was closed using an Amplatzer device.

## Introduction

The vertical vein (VV) may need to be left open or partially ligated in surgical repair of total anomalous pulmonary venous connection (TAPVC). On most occasions, it spontaneously closes later on but it uncommonly remains patent leading to a significant left‐to‐right shunt and requiring closure 1, 2. We describe a case of a recanalized VV discovered in a 7‐year‐old boy having undergone surgical repair of TAPVC in infancy and in whom the discovery of right heart chambers dilation called for further investigations. The left‐to‐right shunt was suppressed by a percutaneous closure of the VV using an Amplatzer Vascular Plug II.

## Case Report

**Figure 1:**
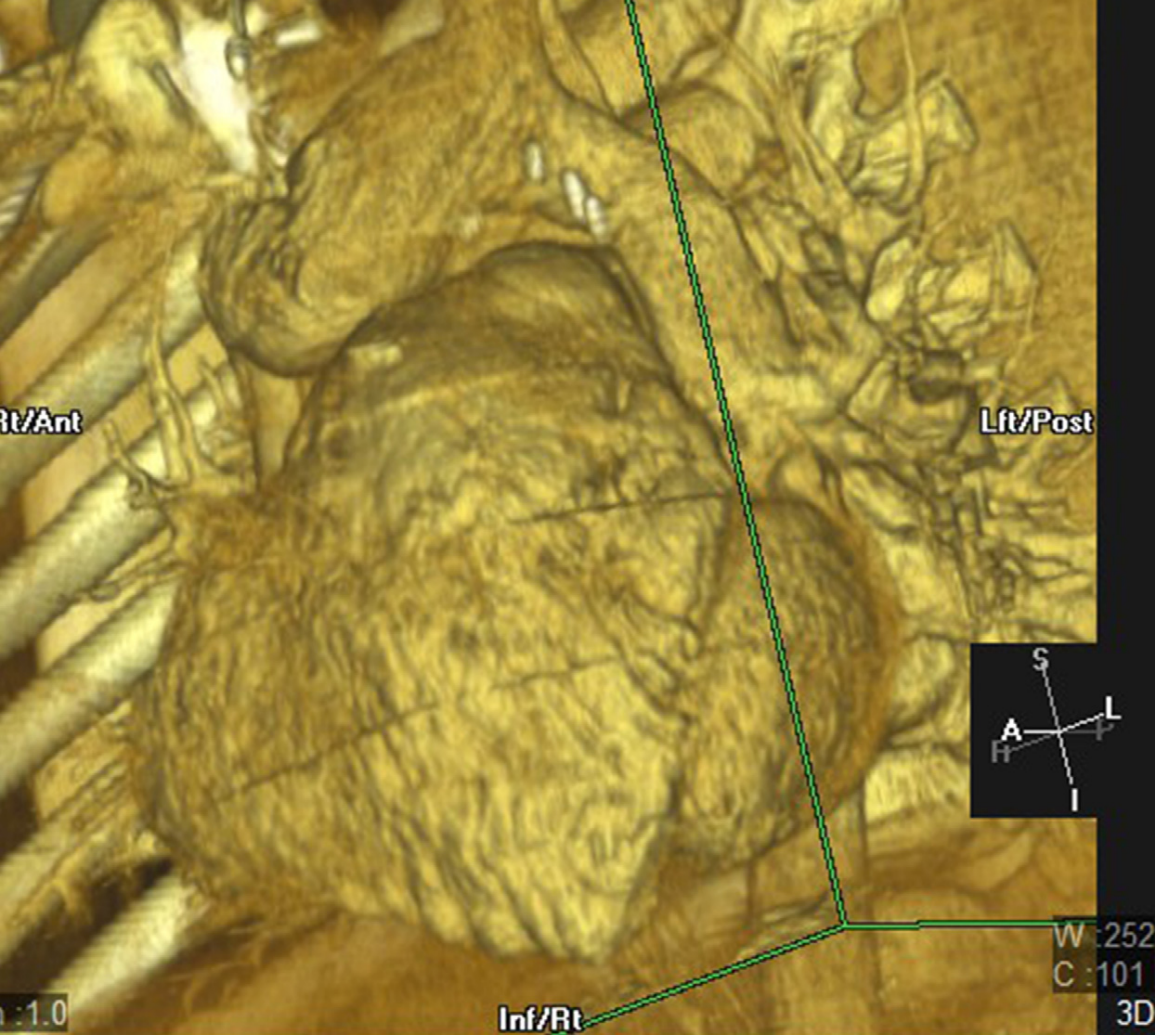
Reconstructed 3D image of a computerized tomography showing the VV and the neighboring vessels.

A 7‐year‐old 25 kg male was admitted to our care after follow‐up echocardiography with Doppler showed dilation of the right heart chambers and of the superior vena cava. This patient was diagnosed at the age of 1 month with supracardiac TAPVC after having presented with respiratory distress, cyanosis, and heart murmur. He was uneventfully operated at 45 days of life of total surgical repair including redirection of the pulmonary venous return (PVR) drainage to the left atrium and atrial septal defect closure. The VV was partially ligated at the level of its connection with the left Innominate vein (LIV). He was then lost from our care and came back to our attention at the age of 7 years for progressive fatigue appearing on moderate effort. Physical examination showed no heart murmur, and oxygen saturation measured on room air was around 99%. Echocardiography with Doppler showed no intracardiac shunt and moderate‐to‐severe right heart chambers dilation as well as dilation of the superior vena cava. The QP/QS was estimated at 2.5/1. A multidetector 64‐slice computerized thoracic scan was performed and showed a recanalization of the VV connecting the PVR to the LIV (Fig. 1). The VV segment was 3 cm long and 15–17 mm large. This segment was elected as a target “landing zone” for an endovascular occluding device.

## Technique

**Figure 2:**
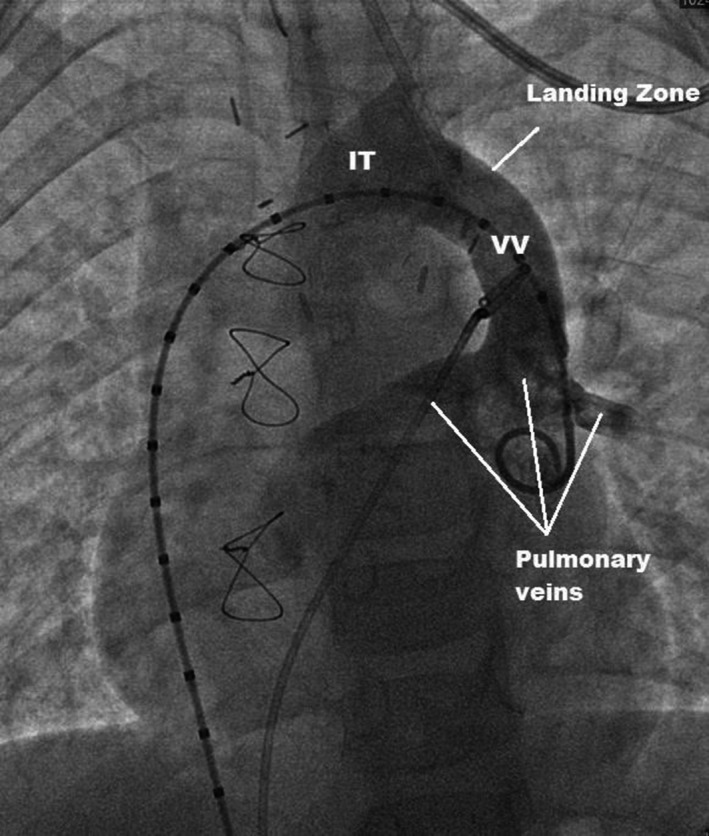
Dye injection in the VV showing a 4‐ to 5‐cm‐long, 15‐mm‐wide segment (landing zone) between the LIV (hereby referred to as IT, innominate trunk) and the pulmonary veins.

**Figure 3:**
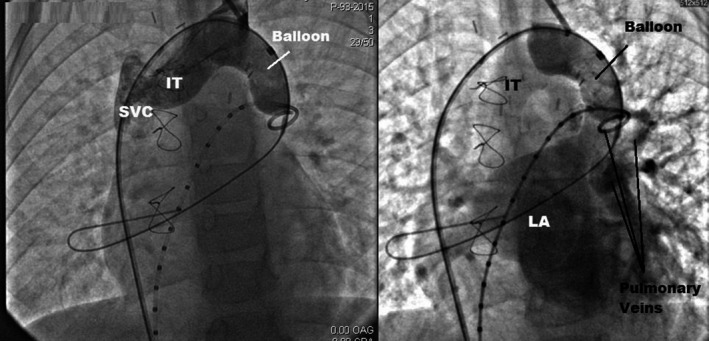
Contrast injection in the left jugular vein (left) and in the pulmonary artery (right) while ballooning the landing zone showing the accurate location of this zone and confirming the patency of both the pulmonary return and the junction between the left jugular vein and the LIV (hereby referred to as IT, innominate trunk).

**Figure 4:**
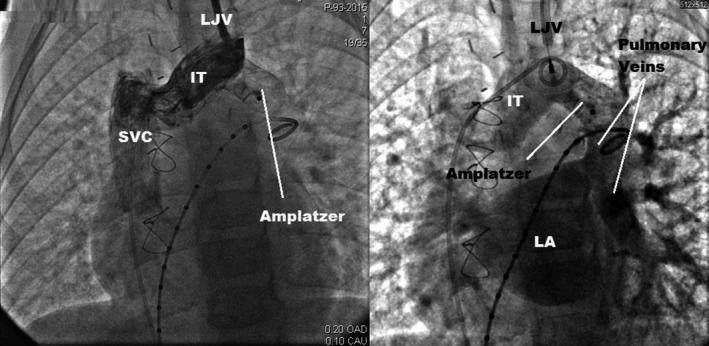
Contrast injection in the left jugular vein (left) and in the pulmonary artery (right) after the detachment of the occluding device confirming the separation of the systemic and pulmonary venous systems and the patency of all neighboring vessels.

Written consent was obtained from the parents prior to the procedure. Under general anesthesia, both femoral veins and the left internal jugular vein were cannulated. The pulmonary pressure was normal, and the QP/QS was calculated at 2.7/1. A 2‐mmHg systolic gradient was detected between the proximal collector and the LIV. A 5‐Fr calibrated pigtail catheter was inserted from the right femoral vein to the VV through the LIV. The first angiography performed in the VV showed drainage from the PVR into the VV and the LIV (Fig. 2). Another pigtail catheter was introduced into the pulmonary artery through the left femoral vein. Pulmonary angiography showed the location of all PVR drainage on the lower part of the VV. The target “landing zone” of the VV was 15 mm large and 35 mm long. A 24‐mm‐large and 45‐mm‐long Amplatzer^®^ Sizing Balloon II was introduced through the right femoral vein in a 9‐Fr short introducer, advanced on an exchange wire to the “landing zone,” and then inflated with diluted contrast media for total occlusion. Separation of the pulmonary and systemic venous returns was thus momentarily obtained, while respecting the drainage of the pulmonary vein to the left atrium and the continuity between the left jugular vein and the LIV. This was checked by contrast injections in the left internal jugular vein and the pulmonary artery (Fig. 3). Ballooning the VV was also helpful for the accurate location of the landing zone segment and for calibration of the vessel diameter. As previous angiography measures were confirmed with the balloon, we decided to close the VV using a 22‐mm Amplatzer Vascular Plug II. The device was introduced in a 9‐Fr, 11‐cm sheath inserted in the left internal jugular vein. Before device detachment, the separation as well as the patency of both circulations were tested again. The device was uneventfully detached, and patency as well as separation were also documented (Fig. 4). The child was discharged from the hospital on the next day. Six months following the procedure, echocardiography showed no heart chamber dilatation and a normal biventricular systolodiastolic function.

## Discussion

Total anomalous pulmonary venous connection (TAPVC) is a relatively uncommon congenital defect in which the pulmonary veins connect with the right atrium instead of the left atrium, either directly or by an alternative pathway 3, 4. The diagnosis of TAPVC is an indication for surgery. If the condition remains untreated, it is usually fatal before 1 year of age 4. Repair of TAPVC involves anastomosing the pulmonary venous confluence with the left atrium and ligating the vertical vein 5. Whether the VV has to be left open or ligated during repair of a TAPVC is still controversial 6. A patent VV can unload the small, noncompliant, left‐sided cardiac chambers by functioning as a temporary reservoir for pulmonary venous blood after TAPVC repair 6. On most occasions, the vertical vein spontaneously closes after surgery due to preferential flow to the left atrium as its compliance improves. However, there have been instances where the unligated or partially ligated vertical vein remained patent, which in turn leads to a significant left‐to‐right shunt requiring surgical ligation or preferably device closure 1, 2.

In our patient, right heart chambers dilatation in the absence of interatrial residual shunt on echocardiography raised the suspicion of persistent VV drainage in the systemic venous return. The angioscan was the cornerstone of diagnosis detailing the anatomy of the VV. During the procedure, balloon testing was a necessary step before the final device closure. It helped us confirm the vessel diameter and length. Additionally, it clearly defined the target landing zone, confirming the patency of PVR to the left atrium and the left internal jugular vein to the LIV during balloon occlusion (Fig. 3) 7. The occluding device is generally selected according to the size, anatomy, the presence of tapered segments and the hemodynamic characteristics of the vascular malformation. For this purpose, the Amplatzer^®^ (St. Jude Medical, Inc., Minnesota, USA) Vascular Plug II was the optimal choice for this large and relatively short low‐pressure segment. In addition, delivering the device from the left internal jugular vein allowed a straight and easy access for its implantation. Percutaneous occlusion of the VV was the optimal choice in this case given the risks of a redo surgery and the advances in cardiac catheterization.

The interaction between surgery and interventional cardiology leads to incessant improvement in medical and surgical outcomes. Hybrid procedures manifest the best example of this fruitful collaboration. While the necessity of surgical “backup” is well documented for interventional cardiology in structural heart defects, the role of the interventionist in bailing out surgical drawbacks is still underestimated. We hereby report an example of avoiding redo surgery in a small boy using a state‐of‐the‐art catheter technique. Combining different imaging tools was essential to accurately delineate the anatomy, thus optimizing the final result.

## Authorship

RC: gathered the patient data, performed a literature review, and wrote the manuscript. NH and ZS: performed the procedure. LD: is the referring physician; she performed the ultrasounds and did the follow‐up.

## Conflict of Interest

None declared.
